# A Large Language Model-Guided IMU-PDR Method for Detection and Error Correction

**DOI:** 10.3390/s26185897

**Published:** 2026-09-17

**Authors:** Guowei Liang, Hongyu Wu, Siqi Bai, Hong Tang

**Affiliations:** 1 School of Engineering, Sichuan Normal University, Chengdu 610021, China; 2023180127@stu.sicnu.edu.cn (G.L.); 20242195018@stu.sicnu.edu.cn (H.W.); 20160093@sicnu.edu.cn (H.T.); 2 National Key Laboratory of Science and Technology on Blind Signal Processing, Chengdu 610000, China

**Keywords:** pedestrian dead reckoning (PDR), large language model (LLM), chain-of-thought reasoning (CoT)

## Abstract

In environments where global navigation satellite system (GNSS) signals are unavailable or unreliable, inertial measurement unit (IMU)-based pedestrian dead reckoning (PDR) offers considerable potential because it operates independently of external infrastructure. However, maintaining stable and accurate positioning across varying conditions remains challenging. To address this issue, this paper proposes a large language model (LLM)-guided IMU-PDR framework for detection and error correction. The proposed method transforms IMU time-series data into a structured representation tailored to PDR tasks and incorporates domain-specific prior knowledge into prompts to guide the LLM in step-count detection, step-length estimation, heading correction, and subsequent trajectory reconstruction. Experimental results demonstrate that the proposed framework improves step-count detection accuracy, achieves competitive step-length estimation performance, effectively suppresses heading drift, and enhances the overall consistency of reconstructed trajectories. These results suggest that, when guided by domain-specific prior knowledge, LLMs have the potential to interpret and reason effectively over IMU signals, providing a new approach to intelligent detection and error correction in IMU-PDR.

## 1. Introduction

With the growing demand for continuous and reliable localization, pedestrian positioning has become increasingly important in applications such as indoor navigation, emergency rescue, health monitoring, and smart wearable devices. In indoor environments, underground spaces, and dense urban areas, external positioning signals are often obstructed or severely attenuated, which may lead to signal degradation or complete loss of positioning availability [1,2]. As a result, conventional satellite-based localization methods often struggle to provide continuous and stable positioning. In contrast, inertial measurement unit (IMU)-based pedestrian dead reckoning (PDR) relies on sensors such as accelerometers and gyroscopes to continuously capture pedestrian motion, enabling autonomous localization without dependence on external infrastructure. Therefore, IMU-based PDR has emerged as an important approach for continuous pedestrian localization in environments where external positioning signals are unavailable or unreliable.

In recent years, large language models (LLMs), exemplified by ChatGPT, have achieved substantial advances and significantly accelerated the development of natural language processing. Benefiting from their strong capabilities in semantic representation, knowledge utilization, and complex reasoning, LLMs have demonstrated considerable strengths in natural language understanding and generation, human-like interaction, knowledge-intensive task processing [3], and prompt-based reasoning [4]. However, because LLMs are primarily trained on textual data, their ability to interpret and reason over physical sensor signals, such as IMU data, remains relatively limited. Compared with purely textual inputs, IMU signals exhibit stronger temporal dependencies and physical constraints, while encoding gait rhythms, motion characteristics, and directional changes. Therefore, effectively integrating domain-specific prior knowledge with LLM-based reasoning for key parameter estimation and error correction in PDR remains an important research challenge.

A typical IMU-PDR system comprises three core modules: step detection, step-length estimation, and heading estimation.

Step detection is a fundamental component of PDR and provides the basis for subsequent step-length estimation and trajectory reconstruction. Common step-detection approaches include peak detection [5], zero-crossing detection [6], frequency-domain methods [7], and zero-velocity update techniques [8]. Among these, peak-detection methods are widely adopted in pedestrian dead reckoning because of their simple implementation, low computational complexity, and suitability for real-time applications. Xu et al. [9] proposed an adaptive threshold-updating mechanism based on peak detection. By dynamically adjusting the peak-detection threshold and incorporating a time-interval constraint, the method improved step-detection performance under unconstrained carrying conditions. Shi et al. [10] introduced multiple criteria, including periodicity, gait similarity, and continuity constraints, within an adaptive peak-valley threshold framework, thereby enhancing detection robustness to smartphone posture changes and non-walking interference. Bai et al. [11] proposed an adaptive time-threshold algorithm based on the estimated gait period (ATT-EGP). The method estimates the average gait period through peak-valley pairing, dynamically adjusts the temporal threshold used for peak screening, and combines amplitude thresholds with peak-selection regions to eliminate pseudo-peaks, thereby improving step-detection performance across different motion states, including walking, brisk walking, and running.

To enhance gait recognition performance and step-detection accuracy, Zhang et al. [12] proposed an improved finite state machine (IFSM)-based gait detection method. The method divides the acceleration pattern within a single step into five states and determines the step count by combining adjacent acceleration differences with a noise-masking mechanism. Vandermeeren et al. [13] proposed a deep learning-based step-boundary detection method that employs a long short-term memory (LSTM) network to identify the start and end points of individual steps from acceleration sequences, thereby enabling more accurate step detection.

Thio et al. [14] further proposed a continuous gait detection method based on sinusoidal approximation, which supports continuous real-time updating by identifying both complete and fractional steps, thereby enhancing localization continuity in PDR. Overall, these methods can improve step-detection accuracy to a certain extent. However, in practical applications, they often still depend on relatively complex signal-processing pipelines, manually tuned parameters, or additional model training, which may limit their adaptability and practical usability.

Step-length estimation plays a crucial role in position updating in PDR. Conventional step-length estimation methods include constant models, linear models [15,16,17], nonlinear models [18,19,20], and neural network-based approaches [21,22,23]. Constant and linear models are computationally efficient but often exhibit limited robustness under varying conditions, such as inter-individual differences and changes in walking speed. Neural network-based models offer stronger nonlinear modeling capabilities and improved robustness; however, their relatively high computational cost and implementation complexity may hinder practical deployment. In comparison, nonlinear models can better capture the complex relationship between step length and IMU-derived parameters through feature mapping and can further enhance estimation robustness through adaptive coefficient updating [24].

Heading estimation is a key step affecting trajectory reconstruction accuracy. Typical methods include Kalman filtering [25], multisensor fusion [26,27], machine learning-based methods [28], and heuristic drift elimination (HDE) [29]. Although these methods can improve heading estimation accuracy to some extent, they may still suffer from high algorithmic complexity, strong dependence on environmental conditions, and insufficient global constraints. In contrast, geometric prior-based methods [30] exploit spatial regularities in trajectories, such as directional continuity, turning characteristics, and parallel or orthogonal relationships, to perform global adjustment of heading drift. Such methods can achieve accurate heading estimation with relatively low computational overhead while improving the geometric consistency of reconstructed trajectories. In recent years, various drift-resistant heading estimation and inertial positioning enhancement methods have been proposed to address accumulated heading drift caused by low-cost inertial sensors. Mansour et al. [31] proposed an adaptive calibration method combining Wi-Fi fingerprinting and magnetic stability information, in which reliable environmental information is introduced to assist heading calibration and reduce accumulated heading drift. Ye et al. [32] proposed a deep-learning-assisted heading estimation framework that integrates smartphone inertial measurements with visual structural information and uses the extracted structural features to assist in constraining heading estimation, thereby improving the robustness of heading estimation. In addition, Zhang and Xu [33] proposed an arithmetic optimization-based PDR method for smartphones, which optimizes key PDR parameters and establishes a heading correction mechanism according to pedestrian motion states to enhance the accuracy and robustness of pedestrian positioning. These studies have improved PDR robustness and positioning performance from different perspectives, including adaptive calibration, multisensor information fusion, learning-based heading enhancement, and parameter optimization.

As the capabilities and scope of LLM applications continue to expand, LLM applications have extended from traditional natural language processing tasks to multimodal information understanding [34], structured data processing [35], human activity recognition [36], and engineering applications. Although LLMs still have a limited ability to directly understand physical sensing signals, recent studies have begun to explore their use in sensing-related tasks. Hota et al. [37] used LLMs as virtual annotators to annotate encoded sensing data, whereas Yu et al. [38] combined sensing signals with LLM-based reasoning to develop a health-prediction method. These studies demonstrate the potential of integrating LLMs with physical sensing information; however, their application to PDR detection and error correction remains insufficiently explored.

To address this research gap, this paper proposes an LLM-guided IMU-PDR method for detection and error correction. Unlike conventional text-oriented applications of LLMs, the proposed method targets continuous motion data acquired by IMUs and incorporates domain-specific prior knowledge to guide the LLM in performing key PDR tasks, including step-count detection, step-length estimation, and heading correction, thereby supporting subsequent PDR trajectory reconstruction. The main contributions of this study are summarized as follows.

First, a task-oriented data-representation and prompting strategy is developed to transform processed IMU time-series data into structured inputs suitable for different PDR tasks and to incorporate the corresponding domain-specific knowledge into the prompts.

Second, a Chain-of-Thought Iterative Bias Compensation (CIB) method is proposed for heading-drift correction. Using the stepwise heading sequence and the initial reconstructed trajectory as inputs, the CIB method identifies motion structures and drift characteristics and iteratively evaluates and refines candidate compensation parameters based on the corresponding corrected trajectories, thereby determining an equivalent zero-bias compensation parameter suitable for the current trajectory.

Third, the proposed method is evaluated under controlled walking, natural walking, stop-and-go, pocket-carrying, and comprehensive-path conditions. Its output repeatability and practical processing latency are also examined to provide a more comprehensive evaluation of the proposed method.

## 2. Materials and Methods

### 2.1. Preliminary Knowledge

#### 2.1.1. Fundamentals of Pedestrian Dead Reckoning

**Figure 1:**
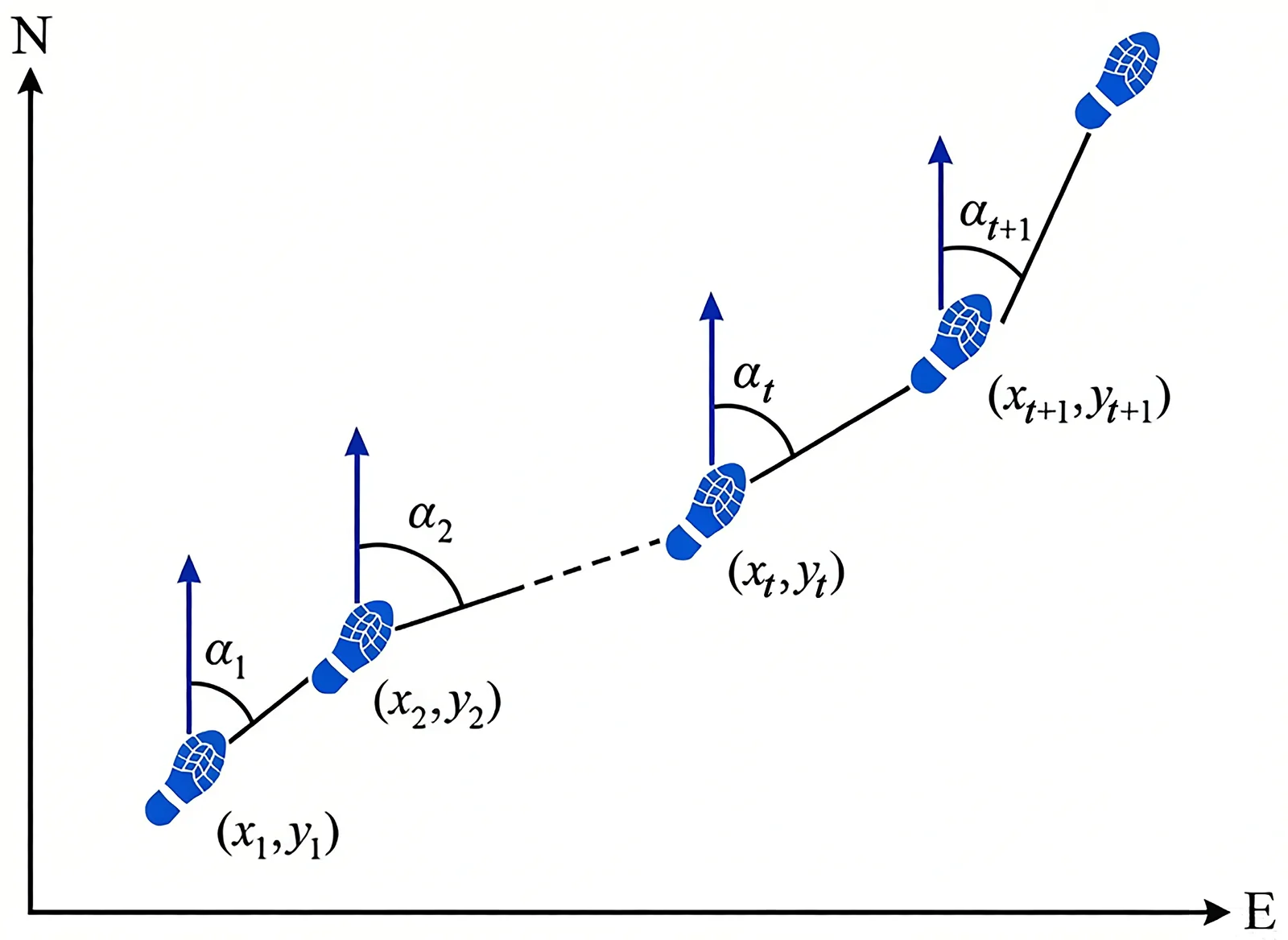
Position update principle of pedestrian dead reckoning (PDR).

Pedestrian dead reckoning (PDR) is an autonomous localization approach that recursively estimates a pedestrian’s position using IMU measurements obtained from sensors such as accelerometers and gyroscopes. Its positioning performance primarily depends on three key parameters: step count, step length, and heading. As illustrated in Figure 1, PDR reconstructs the pedestrian trajectory by continuously updating the displacement and movement direction at each detected step. The position update equations are expressed as follows:

(1) $$\left\{\begin{array}{c} x_{t+1}=x_{t}+L_{t}\cdot \sin\alpha_{t}\\ y_{t+1}=y_{t}+L_{t}\cdot \cos\alpha_{t} \end{array}\right.,$$

where $x_{t+1}{\;\mathrm{and}\;y}_{t+1}$ denote the x- and y-coordinates of the positioning point at step t+1, respectively, which are determined by the previous positioning point ($x_{t}{,y}_{t}$), the corresponding step length $L_{t}$ and the heading angle $\alpha_{t}$.

#### 2.1.2. IMU-Based Pedestrian Dead Reckoning Method

**Figure 2:**
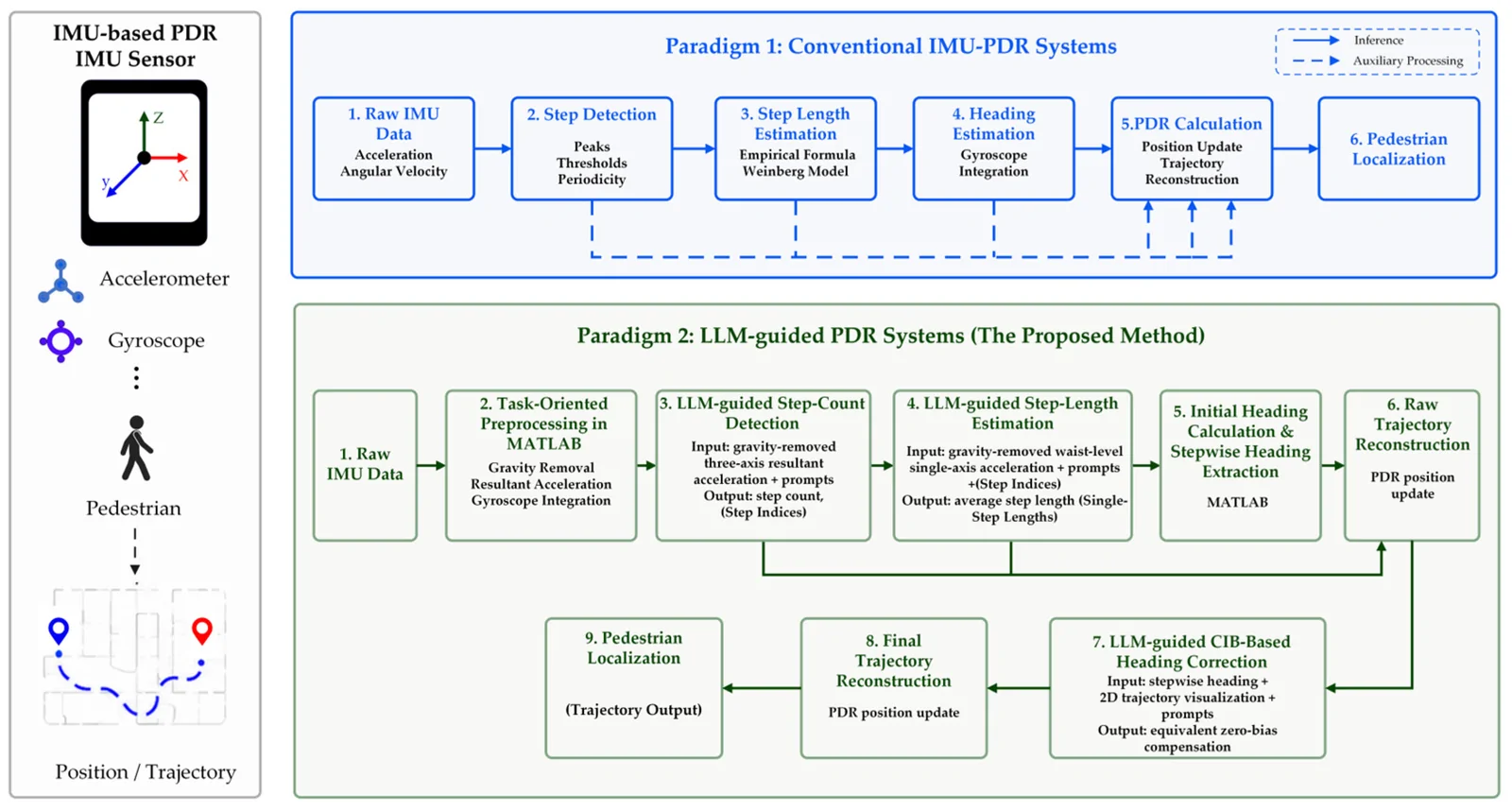
Implementation flow of the two PDR paradigms.

In practical IMU-PDR systems, raw IMU data typically undergo step detection, step-length estimation, and heading estimation before being used for trajectory reconstruction. Based on how these key parameters are processed, this paper considers two paradigms, as illustrated in Figure 2.

Paradigm 1: Conventional IMU-PDR Systems. Conventional IMU-PDR systems typically employ separate algorithms for different processing tasks. Specifically, step detection mainly relies on peak characteristics, threshold criteria, and periodic patterns in acceleration signals; step-length estimation is commonly performed using empirical models, such as the Weinberg model; and heading is primarily estimated by integrating gyroscope measurements. Once these key parameters are obtained, pedestrian dead reckoning can be performed to reconstruct the walking trajectory.

Paradigm 2: LLM Reasoning-Based PDR Systems. In this paradigm, the inputs consist of PDR-task-oriented structured data and carefully designed prompts, which together support pedestrian localization. Domain-specific prior knowledge is incorporated into the prompts to guide the LLM in interpreting IMU data, performing reasoning, and estimating key parameters required for PDR. In this way, the LLM assists in completing key tasks such as step detection, step-length estimation, and heading correction. Compared with conventional systems, the LLM-guided paradigm reduces reliance on complex signal-processing pipelines and extensive parameter tuning, thereby providing a potentially more flexible and adaptive approach to pedestrian dead reckoning.

### 2.2. Methodology

#### 2.2.1. Overall Data Processing and LLM Intervention Workflow

After IMU data acquisition is completed for each experimental trial, the raw three-axis acceleration and angular velocity time series are processed in a task-oriented manner according to the requirements of the corresponding PDR tasks. The processed data are then provided to the LLM together with the associated domain-specific knowledge prompts.

For the step-count detection task, the three-axis acceleration data are processed to remove the gravitational component and construct a gravity-removed three-axis resultant acceleration sequence, which is then provided to the LLM as a numerical time series. Guided by the criteria specified in the prompt, including periodicity, prominence, rhythmicity, and peak-shape consistency, the LLM identifies genuine step peaks. During step-count performance evaluation, the LLM outputs only the final step count for the complete experimental segment. During trajectory reconstruction, the index corresponding to each detected step is additionally obtained to determine its temporal location within the IMU time series.

For the step-length estimation task, the gravity-removed waist-level single-axis acceleration time series is used as the primary input. The sampling rate, sensor placement, and subject height are incorporated as physical-scale constraints to guide the LLM in analyzing gait-cycle characteristics and motion intensity and in estimating the average step length. To generate the single-step length sequence required for trajectory reconstruction, the detected step indices are further combined with the average step-length constraint to estimate step-specific lengths consistent with the motion characteristics of individual steps.

For heading processing, the gyroscope angular velocity data are first integrated in MATLAB (vR2021b) to obtain a continuous initial heading sequence. The heading corresponding to each detected step is then extracted using the same step indices, thereby ensuring a one-to-one correspondence between the single-step length sequence and the stepwise heading sequence. The raw trajectory is subsequently reconstructed according to the PDR position update equations.

During heading correction using the CIB method, the stepwise heading sequence in .mat file format, the two-dimensional trajectory visualizations generated by MATLAB, and the domain-specific knowledge prompts corresponding to different reasoning stages are provided to the LLM. Guided by the incorporated domain knowledge, the LLM sequentially performs motion-structure identification, drift-pattern analysis, candidate compensation parameter comparison, and final equivalent zero-bias compensation parameter selection. The selected compensation parameter is then used to correct the entire heading sequence, and the final trajectory is reconstructed from the single-step lengths and corrected stepwise headings according to the PDR position update equations.

In this study, the complete time series from each individual experimental trial is used as the basic input unit for the LLM. Accordingly, the input length depends on the duration of the corresponding experimental segment, and the time series is not segmented using fixed-length or sliding windows. Depending on the requirements of different PDR tasks, the processed time series for step-count detection and step-length estimation is stored as a numerical sequence in .txt format and provided to the LLM together with the corresponding domain-specific knowledge prompts. For CIB-based heading correction, the stepwise heading sequence is provided in .mat format, while the two-dimensional trajectories generated by MATLAB are supplied as trajectory visualizations. Representative examples of the input formats used for different PDR tasks are provided in Appendix B. Deterministic numerical processing procedures, including gravity removal, three-axis resultant acceleration calculation, gyroscope integration, and PDR trajectory reconstruction, are implemented in MATLAB, whereas the domain-specific knowledge prompts guide the LLM in task-specific analysis, reasoning, and parameter determination.

Overall, the processing workflow comprises IMU data preprocessing, step-count detection and step-index extraction, step-length estimation and single-step length generation, initial heading calculation, raw trajectory reconstruction, CIB-based heading correction, and final trajectory reconstruction. The inputs, processing procedures, and outputs at each stage are further illustrated in the corresponding workflow diagram.

#### 2.2.2. Domain Knowledge and Prompting Rationale for Key PDR Tasks

Step-Count Detection Knowledge. During normal walking, acceleration signals typically exhibit clear periodic patterns, with approximately consistent intervals between successive step peaks. An excessively short interval between two peaks may indicate a pseudo-peak or noise, whereas an excessively long interval may suggest a missed step. In addition, the amplitude of a genuine step peak is generally significantly higher than the local baseline, providing an important criterion for distinguishing valid step events from local disturbances.

Step-Length Estimation Knowledge. Step length is closely associated with individual characteristics, including height, gait pattern, and walking speed. During normal walking, step length typically varies smoothly and is strongly correlated with step frequency and the amplitude of acceleration fluctuations.

Heading-Correction Knowledge. In IMU-PDR, heading is typically estimated by integrating gyroscope measurements. However, the resulting heading estimate is susceptible to gyroscope bias, sensor noise, and attitude estimation errors, causing heading errors to accumulate progressively over time. During normal walking, these errors can be mitigated by jointly analyzing heading variation patterns and the geometric characteristics of the reconstructed trajectory.

#### 2.2.3. Prompt Design for Step-Count Detection

The step-count detection task takes the gravity-removed three-axis resultant acceleration sequence as input, with the objective of identifying genuine step peaks and determining the final step count. During normal walking, the gravity-removed resultant acceleration typically exhibits a clear periodic peak-valley pattern, with each genuine step peak generally corresponding to one valid step. Therefore, step counting can be performed by detecting genuine step peaks in the acceleration sequence. Using the three-axis resultant acceleration reduces the sensitivity of single-axis signals to variations in sensor orientation, while gravity removal suppresses the gravitational component and better preserves gait-related dynamic fluctuations. As a result, the processed acceleration sequence is more suitable for step-peak detection. The gravity-removed three-axis resultant acceleration sequence is calculated as follows:

(2) $$a_{sum}\left(i\right)=\sqrt{{\left(\frac{a_{x}\left(i\right)}{g}\right)}^{2}+{\left(\frac{a_{y}\left(i\right)}{g}\right)}^{2}+{\left(\frac{a_{z}\left(i\right)}{g}\right)}^{2}}-1,\;i=1,2,\ldots ,n,$$

where $a_{x}(i),\;a_{y}\left(i\right),\;\mathrm{and}\;a_{z}\left(i\right)$ denote the single-axis accelerations along the x-, y-, and z-axes at the i-th sampling instant, respectively, and n denotes the total number of samples in the acceleration sequence.

In the prompt design, explicit criteria are provided to guide the LLM in identifying gait-related information from the acceleration sequence. Specifically, the prompt specifies that genuine step peaks should satisfy four criteria: periodicity, whereby intervals between adjacent peaks should fall within a reasonable range; prominence, whereby peak amplitudes should be clearly higher than the local baseline; rhythmicity, whereby consecutive peaks should exhibit approximately stable temporal intervals; and peak-shape consistency, whereby genuine peaks should display smooth and complete waveforms. Finally, the LLM is instructed to output only a single integer, thereby minimizing redundant information and facilitating result extraction.

By integrating the input format, task definition, discrimination criteria, and output requirements into a unified prompt, the LLM is guided to identify genuine step peaks based on gait-signal characteristics and thereby perform the step-count detection task.

#### 2.2.4. Prompt Design for Step-Length Estimation

The step-length estimation task takes the gravity-removed single-axis acceleration sequence as input, with the objective of identifying and interpreting gait-cycle characteristics and subsequently estimating the average step length. Under the fixed handheld configuration adopted in this study, the waist-level z-axis acceleration during normal walking can effectively reflect the vertical oscillatory motion of the human center of mass. These periodic variations are closely associated with the intensity of individual steps, thereby providing a basis for step-length estimation through gait-cycle analysis.

In the prompt design, domain-specific knowledge related to step-length estimation is incorporated to guide the LLM in interpreting gait information embedded in the acceleration sequence. Specifically, the waist-level sensor placement is explicitly specified and used as a prior constraint, enabling the LLM to interpret the signal in a manner consistent with the motion characteristics at that location. The sampling rate is also provided so that intervals between samples can be converted into temporal intervals for gait-cycle analysis. The LLM is then guided to identify valid gait cycles and extract representative features that characterize the intensity of individual steps. Based on the relationship between these features and step length, the LLM estimates the corresponding step length. The subject’s height is further provided as a physical-scale constraint to restrict the estimated average step length to a reasonable range. Finally, the LLM is instructed to output only a single numerical value to facilitate result extraction and subsequent statistical analysis.

Through this prompt design, the LLM is guided to identify valid gait cycles from the single-axis acceleration sequence, extract representative features reflecting the intensity of individual steps, and estimate the average step length while incorporating physical-scale constraints such as subject height.

After estimating the average step length, step-level motion parameters are further generated for trajectory reconstruction based on the prompt design. It should be noted that, in the step-count detection accuracy evaluation, the LLM is primarily required to output only the final step count to facilitate error calculation and method comparison. In contrast, during trajectory reconstruction, step indices are additionally required to synchronize the single-step length sequence with the stepwise heading sequence. Specifically, the LLM identifies genuine gait peaks from the gravity-removed three-axis resultant acceleration sequence and outputs both the final step count and the index corresponding to each detected step, thereby specifying the temporal location of each step within the sequence. For single-step length generation, the detected step indices, the gravity-removed waist-level z-axis acceleration sequence, and the LLM-estimated average step length are jointly used as inputs. The prompt explicitly defines the average step length as an overall scale constraint, while step-specific fluctuation features reflecting motion intensity are extracted from the acceleration cycle associated with each step. This process produces a single-step length sequence that is consistent with the motion characteristics of individual steps. Meanwhile, a continuous initial heading sequence is obtained by integrating the gyroscope measurements, and the heading corresponding to each step is extracted using the same step indices, ensuring a one-to-one correspondence between the single-step length sequence and the stepwise heading sequence. The single-step lengths and corresponding stepwise headings are then combined according to the PDR position update equations to reconstruct the raw trajectory, which is subsequently used as the input to the CIB method.

#### 2.2.5. Prompt Design for Chain-of-Thought Iterative Bias Compensation (CIB)

The heading-correction task aims to mitigate cumulative heading drift caused by gyroscope bias, sensor noise, attitude estimation errors, and other error sources, thereby improving localization accuracy. To this end, equivalent zero-bias compensation is introduced to approximately correct the accumulated heading deviation arising from multiple sources of error and to enhance the overall geometric consistency of the reconstructed trajectory. Compared with more complex heading-correction approaches, equivalent zero-bias compensation provides a relatively direct mechanism for reducing heading error. In particular, in environments where external sensing or positioning support is unavailable, it offers an efficient correction strategy without requiring additional infrastructure.

To determine an appropriate equivalent zero-bias compensation parameter for the current experiment, this paper develops a chain-of-thought-based iterative heading-correction method. The method progressively identifies a suitable compensation parameter to reduce cumulative heading error and improve overall localization performance. Chain-of-thought (CoT) reasoning decomposes a complex task into a sequence of interconnected subtasks, in which each reasoning step builds on the results obtained in preceding steps, thereby progressively guiding the model toward the target output. This stepwise reasoning strategy facilitates structured problem solving and provides a more explicit intermediate reasoning process for complex tasks [39]. The reasoning process can be expressed as follows:

(3) $$\mathrm{CoT}\;\mathrm{Process}=f(\mathrm{Input},\{s_{1},s_{2},\ldots ,s_{n}\},\mathrm{Output}),$$

where Input denotes the input data or problem, Output denotes the final output, and {s1, s2, …, sn} represents the sequence of reasoning steps or substeps in the chain of thought. Meanwhile, an iterative strategy is adopted to adaptively adjust the compensation strategy through progressive optimization, ultimately yielding the equivalent zero-bias compensation parameter suitable for the current trajectory.

**Figure 3:**
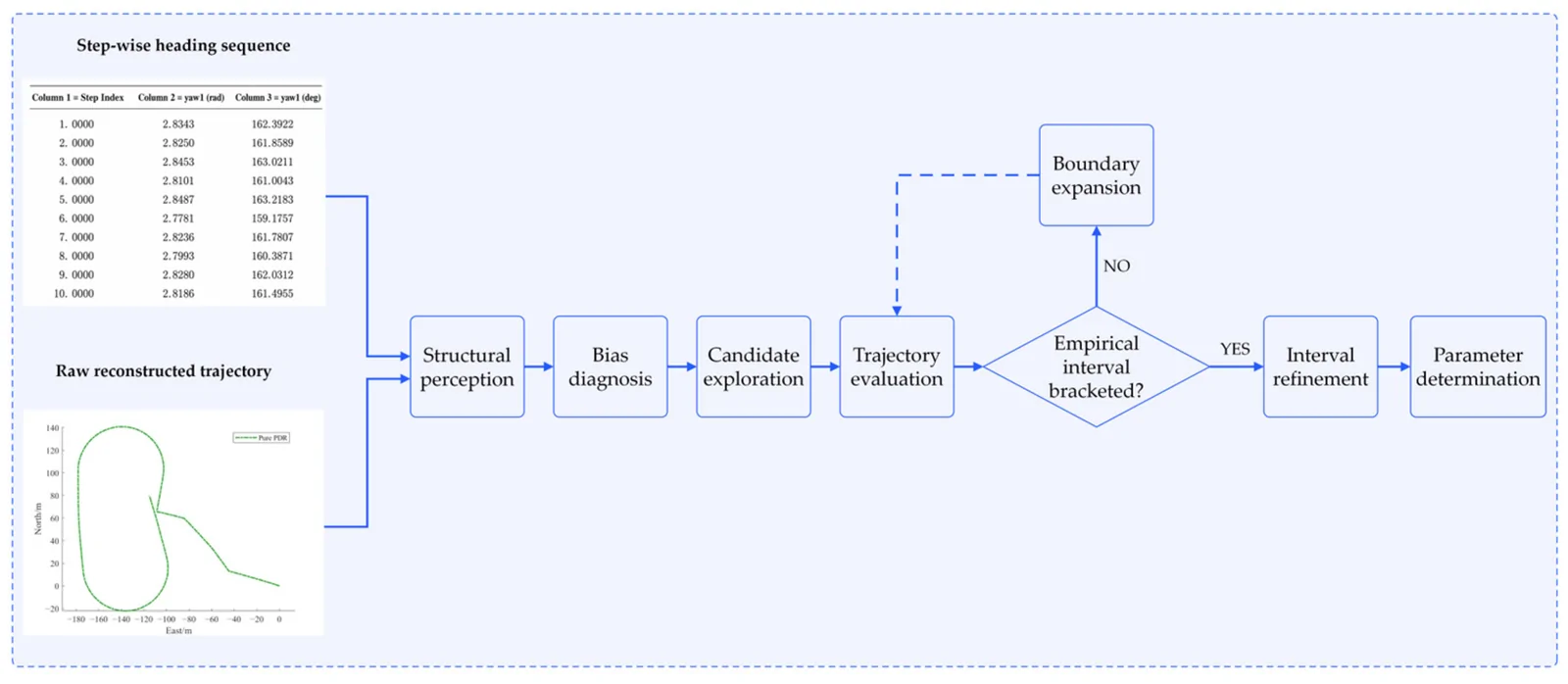
Framework of the proposed Chain-of-Thought Iterative Bias Compensation (CIB) method.

As shown in Figure 3, within the chain-of-thought reasoning process, the stepwise pedestrian heading sequence *Θ* and the raw reconstructed trajectory P are used as inputs to provide the basis for the LLM’s understanding and subsequent reasoning. In the prompt design, the LLM is first guided to identify local motion segments S and infer the most likely global spatial structure type G of the trajectory, thereby enabling an initial perception of the motion state. It is then guided to analyze the drift pattern D caused by residual bias in the current trajectory and to extract the geometric constraints C that should be satisfied by the subsequent compensation, thereby clarifying the evaluation criteria for candidate parameter comparison and the target correction effect. On this basis, the initial zero-bias value $b_{0}$ measured under static conditions is incorporated to guide the LLM in determining the compensation direction δ and generating the initial candidate set B of equivalent zero-bias compensation values, so as to preliminarily define the empirical effective interval I. The static zero-bias value provides a reasonable initial reference range for generating candidate compensation values. Subsequently, by iteratively comparing the corrected trajectory set T corresponding to different candidate parameters, the search range is progressively narrowed, and the candidate parameters are further refined around the locally optimal candidate $b_{\mathrm{best}}$, thereby guiding the LLM to gradually approach the optimal compensation value. Finally, the LLM determines the optimal equivalent zero-bias compensation value $b^{*}$, thereby completing the heading correction task.

To describe the CIB procedure more clearly and enhance the reproducibility of the proposed method, the iterative process is formalized as Algorithm 1, where $B_{I}\;\mathrm{and}\;B_{F}$ denote the set of six candidates within the empirical effective interval and the final set of four candidates, respectively; $T_{I}$ and $T_{F}$ denote the trajectory sets reconstructed using the corresponding candidate compensation parameters, respectively. Compared with the overall framework shown in Figure 3, Algorithm 1 further specifies the sequence of structural analysis, drift diagnosis, candidate generation, boundary expansion, determination of the empirical effective interval, and local refinement, thereby illustrating the logical iterative procedure of the proposed method. In the present proof-of-concept study, this iterative procedure was manually carried out by the researchers through the ChatGPT web interface.

In Algorithm 1, the qualitative judgments involved in motion-structure analysis, drift diagnosis, candidate trajectory comparison, and determination of the empirical effective interval are performed by the LLM based on the corresponding stage-specific prompts and inputs. In contrast, deterministic numerical operations, including the application of candidate compensation parameters, heading correction, and trajectory reconstruction, are performed in MATLAB.
**Algorithm 1.** Iterative search process of the CIB method.Input: Θ, P, b_0_
 Output: b*1: (S, G) ← StructureAnalysis(Θ, P)  2: (D, C) ← DriftDiagnosis(Θ, P, S, G)  3: (δ, B) ← InitialCandidateGeneration(P, S, G, D, C, b_0_)  4: T ← TrajectoryEvaluation(B)  5: while EffectiveIntervalIsUnclear(B, T, C)  do  6:     B ← BoundaryExpansion(B, T, C)  7:     T ← TrajectoryEvaluation(B)  8: end while  9: I ← EffectiveIntervalDetermination(B, T, C)  10: B_I_ ← SixCandidateRefinement(I, T, C)  11: T_I_ ← TrajectoryEvaluation(B_I_)  12: b_best_ ← SixCandidateComparison(B_I_, T_I_, C)  13: B_F_ ← FourCandidateRefinement(b_best_, I)  14: T_F_ ← TrajectoryEvaluation(B_F_)  15: b* ← FinalSelection(B_F_, T_F_, C, I)

By decomposing the complex task into multiple subtasks and combining guided analysis with iterative refinement, the LLM is progressively guided to perform motion-structure recognition, drift diagnosis, and compensation decision-making within a chain-of-thought framework. This process enables the estimation of an appropriate equivalent zero-bias compensation parameter and the subsequent completion of the heading-correction task.

To enhance the reproducibility of the proposed method, representative prompt templates for key tasks, including step-count detection, average step-length estimation, and CIB-based heading correction, are provided in Appendix A.

### 2.3. Experimental Setup

**Figure 4:**
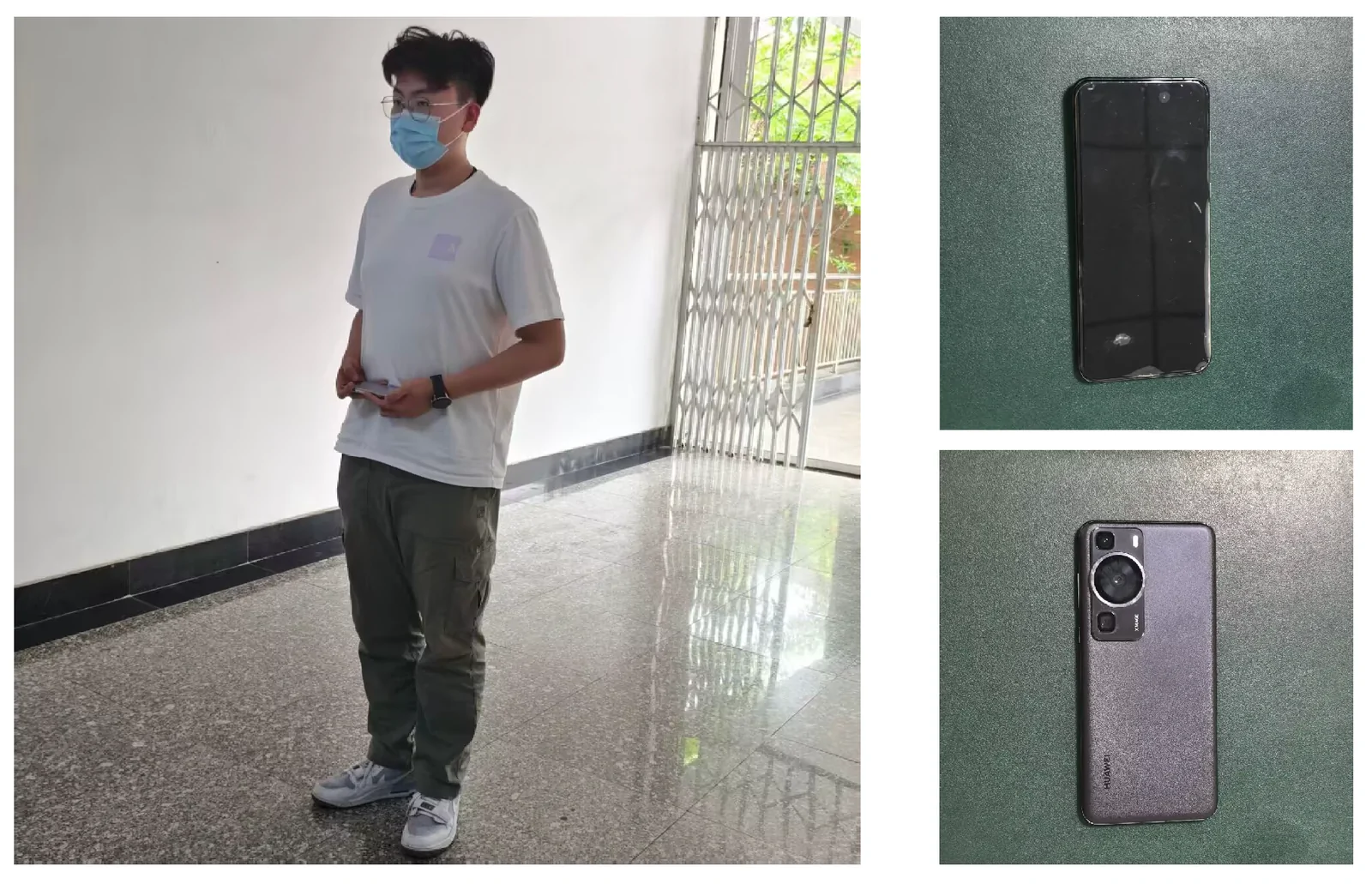
Experimental device and smartphone holding position.

As shown in Figure 4, a HUAWEI P60 (LNA-AL00) smartphone (Huawei Technologies Co., Ltd., Shenzhen, China) was used as the data-acquisition device to collect three-axis acceleration and angular-velocity data from the IMU. During the main experiments, each participant held the smartphone in front of the waist and kept its posture as stable as possible. Because the mobile operating system does not directly expose the underlying sensor chip models, the accelerometer and gyroscope names recognized by the Android system were recorded as ACC S001 004 and GYRO S001 004, respectively. The detailed sensor parameters are listed in Table 1. Three participants with different physical characteristics were recruited for data collection, as listed in Table 2. For each trial, brief stationary segments were recorded before and after walking to support sensor initialization and ensure complete collection of the start- and end-stage motion data. The raw IMU data were then imported into a computer, and all subsequent processing and calculations were performed offline. ChatGPT-5.5 Thinking was used to complete the guided reasoning tasks, and the prompt settings were kept consistent for all tasks of the same type.

The GPT-5.5 Thinking option available through the ChatGPT web interface was used to perform the guided reasoning tasks in this study, including step-count detection, average step-length estimation, and CIB-based heading correction. In this proof-of-concept study, the LLM-guided PDR tasks were conducted manually via the ChatGPT web interface. During the CIB iterative procedure, stage-specific prompts and the corresponding inputs required at each reasoning stage were manually provided to the LLM according to the task requirements. The outputs generated by the LLM were subsequently used for error calculation, method comparison, and trajectory reconstruction. The authors take full responsibility for the accuracy and integrity of the analysis and results.

To clarify the scope and procedures of the experimental evaluation, four categories of experiments were conducted. First, controlled experiments using predefined straight, rectangular, and S-shaped trajectories were performed to evaluate step-count detection and step-length estimation under known geometric and foot-placement constraints. Second, natural-walking experiments were conducted without predefined foot-placement markings to evaluate step-count detection at normal and faster walking speeds and average step-length estimation over a fixed 50 m distance. Third, stop-and-go and pocket-carrying experiments were conducted to examine the effects of changes in motion state and smartphone-carrying configuration on step-count detection. In the stop-and-go experiment, the smartphone was held steadily in front of the waist, whereas in the pocket-carrying experiment, it was placed in the participant’s trouser pocket. Finally, a comprehensive-path experiment was conducted to evaluate CIB-based heading correction. The following subsections describe the objectives, main experimental conditions, reference-value determination, and evaluation methods for the corresponding experiments.

## 3. Results

This section experimentally evaluates the proposed LLM-guided IMU-PDR detection and error-correction method in terms of step-count detection, step-length estimation, heading correction, and output repeatability. The results are organized into controlled-condition validation, natural-walking validation, robustness evaluation under complex walking conditions, comprehensive-path heading-correction evaluation, and repeatability analysis.

### 3.1. Validation Under Controlled Experimental Conditions

To evaluate the basic performance of the proposed LLM-guided method under controlled experimental conditions, step-count detection and step-length estimation were validated using straight, rectangular, and S-shaped trajectory experiments. Before data collection, footfall positions were marked on the ground according to the predefined trajectories and step lengths, and the participants were instructed to complete the walking tasks by following these markings. The manually recorded step counts and the distances between adjacent footfall markings were used as the reference values for step-count detection and step-length estimation, respectively. These three trajectory structures represent unidirectional walking, segmented walking with distinct turns, and continuous directional changes, respectively, allowing the performance of the proposed method to be examined under different motion structures. The corresponding step-count detection and step-length estimation results are presented in the following subsections.

#### 3.1.1. Step-Count Detection Experiment

To evaluate the step-count detection performance of the proposed LLM-guided method under controlled experimental conditions, experiments were conducted using straight, rectangular, and S-shaped trajectories. Manually recorded true step counts were used as the evaluation benchmark. To reduce the influence of experimental randomness, each experiment was repeated several times, and the average result across multiple trials was used for comparison. Three baseline methods were selected for comparison: peak-valley pairwise detection (PVP), fixed double-threshold peak detection (FDT), and ATT-EGP [11]. The relative step-count error was used as the evaluation metric and is defined as follows:

(4) $$E_{N}=\frac{\left|N_{\mathrm{est}}-N_{\mathrm{true}}\right|}{N_{\mathrm{true}}}\;\times \;100\%,$$

where $N_{\mathrm{est}}$ denotes the average number of steps detected by the algorithm, $N_{\mathrm{true}}$ denotes the true number of steps obtained from manual recording or experimental calibration, and $E_{N}$ denotes the relative error of step detection. The experimental results are shown in Table 3.

As shown in Table 3, the LLM-guided step-count detection method achieved low step-count errors across the three participants and three typical trajectories, with an error range of 0.00–4.07% and an average error of 2.84%. By comparison, the peak-valley pairwise detection method produced errors ranging from 1.22% to 42.27%, with an average error of 11.64%; the fixed double-threshold peak detection method produced errors ranging from 1.00% to 9.15%, with an average error of 4.57%; and ATT-EGP produced errors ranging from 0.50% to 47.15%, with an average error of 7.89%. Although the proposed method did not achieve the minimum error in every experimental group, it achieved the lowest average error and lower error variability, demonstrating better detection accuracy and stability across different participants and trajectory structures. The amplitude thresholds used in the conventional peak-detection methods were determined through preliminary experiments. The peak-valley pairwise detection method, fixed double-threshold peak detection method, and ATT-EGP used the same amplitude threshold, Ta = 0.13, whereas the fixed double-threshold peak detection method used a fixed minimum peak distance of Tt = 0.3 s.

**Figure 5:**
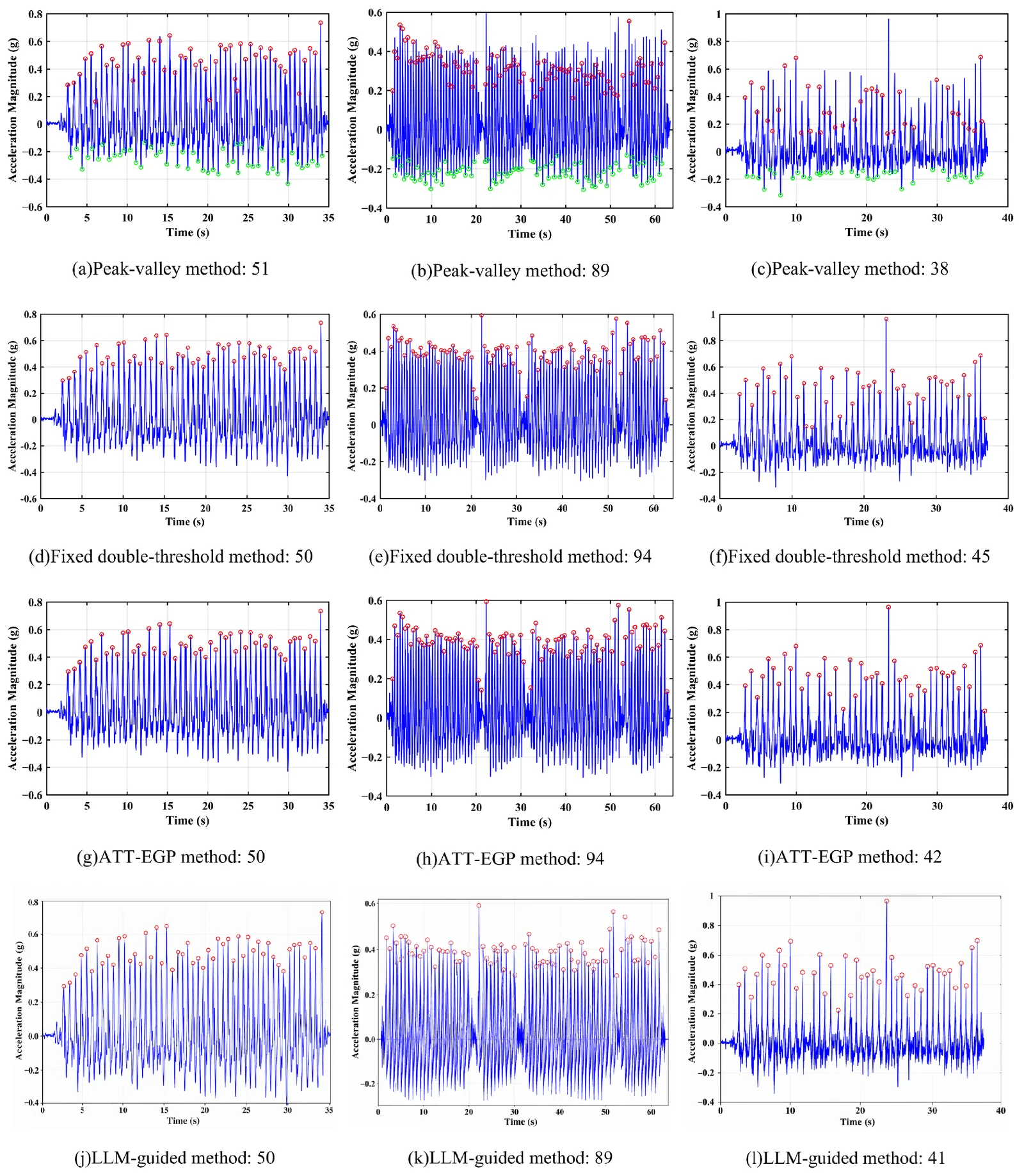
Examples of step-count detection results obtained by the four algorithms under three types of motion trajectories: (**a**–**c**) peak-valley pairwise method, (**d**–**f**) fixed double-threshold peak detection, (**g**–**i**) ATT-EGP method, and (**j**–**l**) LLM-guided method. The blue line in the figure represents the sensor measurement data, while the red and green circles indicate the peaks and troughs detected by the algorithm, respectively.

Figure 5 presents representative step-count detection results obtained by the four algorithms across three types of motion trajectories. As shown in the figure, the straight trajectory exhibits a relatively stable gait rhythm, and all methods achieve comparatively consistent detection performance. In the rectangular trajectory, although turning motions are present, the overall movement is still dominated by segmented straight walking, allowing most methods to maintain satisfactory detection performance. For the S-shaped trajectory, continuous changes in walking direction may disturb the local acceleration waveform and peak–valley structure, thereby increasing the likelihood of missed detections. According to Table 3, in the P3 S-shaped experiment, PVP and ATT-EGP detected averages of only 23.67 and 21.67 steps, respectively, compared with the reference value of 41 steps. For PVP, this undercounting may be associated with reduced peak–valley amplitudes or irregular local structures that prevented some genuine step peaks from forming valid peak–valley pairs. Because ATT-EGP also relies on local peak–valley information to estimate the average gait period and screen candidate peaks, the waveform disturbances may have affected both gait-period estimation and subsequent peak selection. Overall, the LLM-guided step-count detection method demonstrates more consistent performance across different trajectory structures.

#### 3.1.2. Step-Length Estimation Under Controlled Experimental Conditions

To evaluate the step-length estimation performance of the proposed LLM-guided method under controlled experimental conditions, experiments were conducted using the same straight, rectangular, and S-shaped trajectories. The experimental procedure was consistent with that of the step-count detection experiment described above. A height-based proportional model (HBPM) and the Weinberg nonlinear step-length model (Weinberg) were used for comparison. The height-based proportional model is expressed as follows:

(5) $$L_{i}=kH,$$

where $L_{i}$ denotes the estimated step length of the i-th step, H denotes the participant’s height, and k = 0.415 is an empirical proportionality coefficient. The Weinberg nonlinear step-length model is expressed as follows:

(6) $$L_{i}=K\sqrt[{4}]{a_{max}^{i}-a_{min}^{i}},$$

where $a_{\max}^{i}$ and $a_{\min}^{i}$ denote the maximum and minimum values of the resultant acceleration within the i-th step cycle, respectively, and K is an empirical coefficient used to adjust the mapping relationship between acceleration fluctuation features and the actual step length. To quantitatively evaluate the step-length estimation performance of different methods, the relative error of the average step length is used as the evaluation metric. To obtain the reference step length in the step-length estimation experiment, the predefined step length was measured with a tape measure before the experiment, and consecutive footfall points were marked on a flat ground surface. Participants were instructed to complete the walking tasks according to these predefined markings. The distance between two adjacent footfall markings was used as the reference step length for the corresponding experimental group and was used for subsequent error analysis of the step-length estimation results. The relative error of the average step length is defined as follows:

(7) $$E_{L}=\frac{\left|{\bar{L}}_{est}-{\bar{L}}_{ref}\right|}{{\bar{L}}_{ref}}\;\times \;100\%,$$

where ${\bar{L}}_{est}\;$ denotes the estimated average step length, and ${\bar{L}}_{\mathrm{ref}}$ denotes the predefined average step length. The experimental results are shown in Table 4.

As shown in Table 4, the proposed LLM-guided step-length estimation method achieved relative errors ranging from 0.53% to 4.18%, with an average error of approximately 2.51%. The HBPM achieved an average error of approximately 2.55%, indicating that the proposed method and HBPM provided comparable overall step-length estimation accuracy under the controlled experimental conditions. However, their estimation mechanisms differ. The height-based proportional model relies on a fixed height-based relationship and has difficulty reflecting gait variations and local motion disturbances under different trajectory structures; therefore, its adaptability to motion-state changes is limited. The Weinberg model achieved an average error of approximately 3.44%, with individual errors ranging from 0.00% to 8.86%. In this model, the empirical coefficient K was determined from the straight-walking experiment of each participant and then used for subsequent step-length estimation. Therefore, the relatively low errors observed in some straight-walking cases may partly reflect the dependence of the Weinberg model on empirical parameter calibration under matched conditions. When the trajectory structure changes to rectangular or S-shaped trajectories, turning processes, gait-rhythm variations, and local motion disturbances may alter the peak-valley fluctuation characteristics of acceleration, leading to increased errors. Overall, its estimation performance is relatively sensitive to the empirical coefficient K, which usually needs to be manually specified according to the participant, sensor placement, and experimental scenario. Once the parameter does not match the current gait characteristics, noticeable estimation bias may occur. In comparison, the LLM-guided step-length estimation method can estimate step length based on multiple gait features, reducing its dependence on fixed empirical coefficients and repeated parameter tuning while maintaining competitive estimation accuracy.

### 3.2. Validation Experiments Under Natural Walking Conditions

To further evaluate the performance of the proposed method under less constrained conditions, additional natural walking experiments were conducted. Unlike the controlled experiments described in Section 3.1, no predefined foot-placement points were specified in these experiments. The participants completed the walking tasks using their natural gait while holding the smartphone steadily in front of the waist. A HUAWEI P60 smartphone was used to collect accelerometer and gyroscope data at a sampling frequency of 100 Hz.

Two independent experimental settings were designed for step-count detection and average step-length estimation, respectively. The step-count detection experiments were conducted with a fixed ground-truth number of steps to enable direct performance comparison among different step-count detection methods, whereas the average step-length estimation experiments were conducted over a fixed walking distance to evaluate the average step-length estimation capability under natural walking conditions.

Three participants took part in these experiments. For step-count detection, each participant completed three independent trials under both normal- and fast-walking conditions, yielding 18 trial datasets, and each dataset was processed once by the LLM. For average step-length estimation, each participant completed three independent trials over the fixed 50 m walking distance, yielding nine trial datasets. The number of LLM inference runs per step-length dataset was one for P1, two to three for P2, and one to four for P3, yielding 17 LLM inference runs in total across the nine datasets. For datasets subjected to repeated inference, one of the repeated outputs was selected for subsequent analysis. Further details regarding the rerun and output-selection procedure are provided in Section 4. The detected step counts and step-length values reported in Table 5 and Table 6 are expressed as the mean ± sample standard deviation across three independent physical trials for each participant and condition.

#### 3.2.1. Step-Count Detection Experiments Under Natural Walking Conditions

To evaluate the step-count detection performance of the proposed method under natural walking conditions, the participants walked along a straight path using their usual natural gait. Two walking conditions were considered: normal speed and faster speed, with the ground-truth number of steps fixed at 50 for each trial. During the experiments, the smartphone was held steadily in front of the waist, and the actual number of steps was manually counted and recorded. For the IMU data collected in each trial, step-count detection was performed using the proposed LLM-based method as well as the PVP, FDT, and ATT-EGP methods. The relative step-count error was used as the evaluation metric to analyze the influence of natural gait and changes in walking speed on the performance of the different step-count detection methods. The experimental results are presented in Table 5.

As shown in Table 5, the proposed LLM-based method achieved relative errors ranging from 0.00% to 2.00%, with an average error of approximately 0.56% across the evaluated participants and walking speeds. The sample standard deviations of the detected step counts across the three independently collected datasets under each condition ranged from 0.00 to 1.00 steps, indicating small inter-dataset variation. The PVP method achieved an average error of approximately 7.11%, with individual errors ranging from 0.66% to 29.34% and sample standard deviations ranging from 0.58 to 7.51 steps. The average errors of the FDT and ATT-EGP methods were approximately 2.33% and 2.45%, respectively. The pronounced overcounting observed for P3 using the PVP method under normal walking may be associated with participant-specific waveform fluctuations that caused non-gait local extrema to satisfy the peak–valley pairing criteria. Its improved but still less accurate performance at the faster speed suggests that this deviation was not determined solely by walking speed. Overall, the proposed method achieved the lowest average error and maintained low detection errors and small variation among the three datasets at both normal and faster walking speeds.

#### 3.2.2. Step-Length Estimation Under Natural Walking Conditions

To evaluate the step-length estimation performance of the proposed method under natural walking conditions, the participants walked along a straight path using their usual natural gait. The experiments were conducted at a normal walking speed, with the walking distance fixed at 50 m for each trial. During the experiments, the smartphone was held steadily in front of the waist, and the actual number of steps was manually counted and recorded. For the IMU data collected in each trial, step-length estimation was performed using the proposed LLM-based method, the Weinberg method, and the HBPM method. The reference average step length was calculated based on the actual walking distance of 50 m and the corresponding ground-truth number of steps. The relative error of the estimated average step length was used as the evaluation metric to assess the performance of the different step-length estimation methods under natural walking conditions. The experimental results are presented in Table 6.

As shown in Table 6, under natural walking conditions, the proposed LLM-based method achieved relative errors ranging from 0.40% to 0.72%, with an average error of approximately 0.61%. The HBPM achieved an average error of approximately 0.74%, indicating that both methods provided low and comparable average step-length estimation errors, with the proposed method showing a numerically slightly lower value. The sample standard deviations of the LLM estimates across the three independently collected datasets ranged from 0.0044 to 0.0194 m, whereas the zero standard deviations of the HBPM estimates resulted from its fixed output for each participant. The Weinberg method achieved an average error of approximately 4.71%, with sample standard deviations ranging from 0.0043 to 0.0151 m and a relatively large error of 7.98% for P3. Overall, the proposed method maintained relatively stable average step-length estimation performance when the participants walked naturally without following predefined footfall markings, indicating its applicability under the natural-walking conditions evaluated in this study.

### 3.3. Step-Count Detection Under Complex Scenarios

To further evaluate the step-count detection performance of the proposed method under more challenging real-world walking conditions, two experimental scenarios were designed: intermittent walking with stops and pocket carrying. These experiments were conducted to investigate the effects of changes in motion state and device-carrying position on the performance of different step-count detection methods. The specific experimental settings and results are presented below.

Three participants took part in both experimental scenarios, and each participant completed three independent physical trials under each scenario. Consequently, the stop-and-go and pocket-carrying experiments each comprised nine trial datasets. In the stop-and-go experiment, each dataset was processed once by the LLM, resulting in nine LLM inference runs. In the pocket-carrying experiment, all three datasets from P1 were processed once; for P2, one dataset was processed twice, and the other two were processed once; and for P3, two datasets were processed twice, and the remaining dataset was processed once. This resulted in 12 LLM inference runs for the pocket-carrying experiment and 21 runs across the two scenarios in total. For datasets subjected to repeated LLM inference, one output from the multiple inference runs was selected for subsequent analysis. Further details regarding the rerun and output-selection procedure are provided in Section 4. The detected step counts reported in Table 7 and Table 8 are expressed as the mean ± sample standard deviation across the three independent physical trials for each participant. The statistical reporting format and relative-error calculation are consistent with those used in Table 5.

#### 3.3.1. Step-Count Detection Under Intermittent Walking and Stopping Conditions

To evaluate the step-count detection performance of the proposed method under intermittent walking and stopping conditions, the participants walked along a straight path using their usual natural gait, with the ground-truth number of steps fixed at 50 for each trial. During each trial, the smartphone was held steadily in front of the waist. The participants stopped twice at non-predefined positions during walking, remained stationary during each stop, and then resumed walking until the remaining steps were completed. The actual number of steps was manually counted and recorded.

For the IMU data collected in each trial, step-count detection was performed using the proposed LLM-based method as well as the PVP, FDT, and ATT-EGP methods. The relative step-count error was used as the evaluation metric to analyze the influence of alternating walking and stationary states on the performance of the different step-count detection methods. The experimental results are presented in Table 7.

As shown in Table 7, under stop-and-go walking conditions, the proposed LLM-based method achieved relative errors ranging from 1.34% to 3.34%, with an average error of approximately 2.45%. The sample standard deviations of the detected step counts across the three independently collected datasets under each condition ranged from 1.15 to 2.89 steps. The PVP method achieved an average error of approximately 14.00%, with individual errors ranging from 2.00% to 34.00% and sample standard deviations ranging from 2.00 to 7.00 steps, showing particularly large deviations for P3. The average errors of the FDT and ATT-EGP methods were approximately 6.44% and 5.55%, respectively. Intermittent stopping interrupts the periodic gait sequence and introduces transient acceleration fluctuations during deceleration and restarting, which may interfere with peak–valley pairing and threshold-based peak screening. The comparatively small deviations of the proposed method suggest that jointly considering periodicity, prominence, rhythmicity, and peak-shape consistency helped distinguish genuine step peaks from transition-related disturbances under the evaluated stop-and-go conditions.

#### 3.3.2. Step-Count Detection Under Pocket-Carrying Conditions

To evaluate the step-count detection performance of the proposed method when the device-carrying position changes, the participants walked along a straight path using their usual natural gait, with the ground-truth number of steps fixed at 50 for each trial. During each trial, the smartphone was placed vertically in the participant’s trouser pocket and was allowed to move naturally during walking. The actual number of steps was manually counted and recorded. For the IMU data collected in each trial, step-count detection was performed using the proposed LLM-based method as well as the PVP, FDT, and ATT-EGP methods. The relative step-count error was used as the evaluation metric to analyze the influence of changes in device-carrying position and additional motion disturbances within the pocket on the performance of the different step-count detection methods. The experimental results are presented in Table 8.

As shown in Table 8, pocket carrying caused pronounced overcounting for the three conventional methods. The PVP, FDT, and ATT-EGP methods achieved average relative errors of approximately 49.33%, 28.67%, and 54.23%, respectively, with individual errors ranging from 18.00% to 96.00%. Their sample standard deviations across the three independently collected datasets ranged from 0.58 to 6.08 steps, indicating considerable variation under this carrying condition. In contrast, the proposed LLM-based method achieved relative errors ranging from 0.66% to 1.34%, with an average error of approximately 1.11%, while its sample standard deviations ranged from 0.58 to 1.53 steps. Additional smartphone motion inside the trouser pocket may generate local acceleration peaks that are unrelated to genuine gait events but still satisfy the amplitude or temporal criteria used by conventional methods, thereby causing pronounced overcounting. The comparatively small deviations of the proposed method suggest that jointly evaluating periodicity, prominence, rhythmicity, and peak-shape consistency helped reject these pocket-motion-induced disturbance peaks under the carrying configuration evaluated in this study.

### 3.4. Heading-Correction Experiment

To validate the proposed CIB method, heading-correction experiments were conducted along a comprehensive path. Normal walking is one of the most common forms of pedestrian movement in daily life and therefore represents a practically relevant evaluation scenario. Accordingly, the proposed CIB method primarily focuses on correcting heading drift during normal walking. Because normal walking generally exhibits good motion continuity and pedestrian trajectories often contain recognizable geometric structures, a comprehensive path with a regular layout, including straight segments, turning segments, continuous directional changes, and a closed-loop structure, was adopted to evaluate the ability of the CIB method to suppress heading drift and improve PDR trajectory reconstruction. The manually delineated reference route for the comprehensive experiment was derived from Google Earth satellite imagery and saved in KML/KMZ format. This route served as a geometric reference for comparing the reconstructed PDR trajectories.

Four methods are compared in this study: no bias compensation, static bias compensation, traditional HDE, and CIB-based equivalent zero-bias compensation. Under the same initial-heading alignment condition, the no-bias-compensation method directly integrates the raw gyroscope data to estimate the heading and reconstruct the trajectory. The static bias compensation method corrects the gyroscope measurements using the bias estimated during a stationary period. The traditional HDE method applies heuristic heading constraints to correct accumulated heading drift. The CIB-based equivalent zero-bias compensation method uses the equivalent gyroscope bias inferred by the proposed CIB method for heading compensation. Before calculating the route-relative deviation metrics, the reconstructed trajectory was spatially aligned with the manually delineated reference route. Specifically, the starting points of the reconstructed trajectory and the manually delineated reference route were first aligned by translation, after which rotational alignment was performed according to the initial route direction. An additional reference point along the prescribed route was used to determine the appropriate mirrored orientation. No scale adjustment was applied during the alignment process. To establish correspondence between the reconstructed trajectory and the manually delineated reference route, the cumulative arc lengths of the reconstructed trajectory and the manually delineated reference route were normalized to a common path-progress scale. The two paths were then resampled according to the normalized path progress, and the Euclidean distances between corresponding positions were calculated. The resulting pointwise route-relative deviations were further used to calculate the relevant evaluation metrics. To quantitatively compare the reconstructed trajectories obtained using different heading-correction methods, four metrics are calculated from the route-relative deviations: Mean Trajectory Error, RMSE, 95% Error, and Max Error, corresponding to the mean, root-mean-square, 95th-percentile, and maximum values of the route-relative deviations, respectively. These metrics are used to comprehensively evaluate the effectiveness of different methods in improving the geometric consistency of PDR-reconstructed trajectories and suppressing accumulated heading drift.

**Figure 6:**
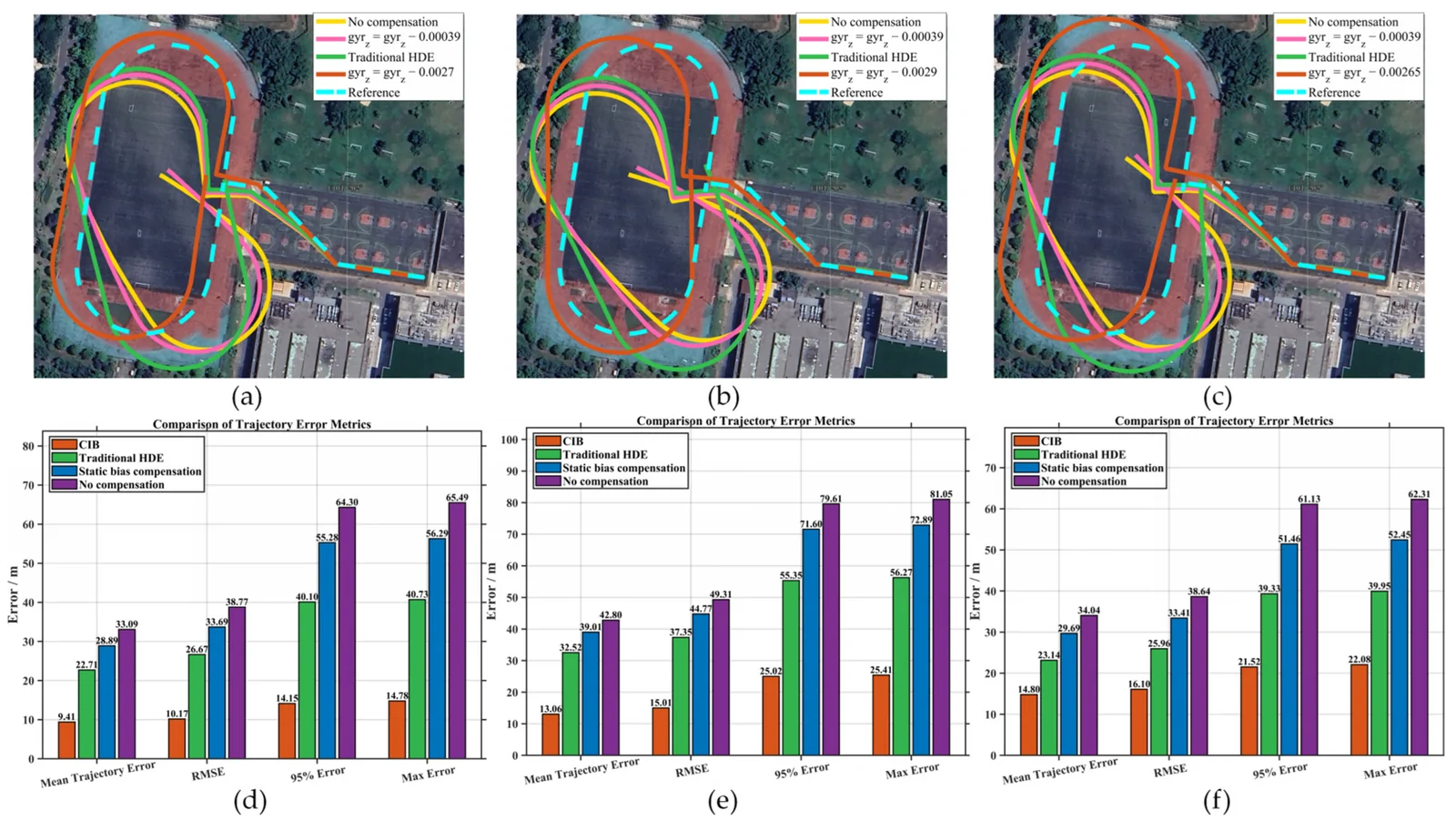
Trajectory reconstruction results and error comparisons along the prescribed walking path under four heading correction methods: (**a**–**c**) trajectory overlay results for the three experimental groups, and (**d**–**f**) comparisons of the corresponding trajectory error metrics.

As shown in the trajectory overlay results in Figure 6, the uncompensated trajectories exhibit obvious overall drift and geometric deformation in all three experimental groups. Static bias compensation reduces the trajectory deviation caused by errors accumulated through raw gyroscope integration to some extent, although its improvement varies across the experimental groups. The traditional HDE method further suppresses part of the accumulated heading drift and improves the agreement between the reconstructed trajectories and the manually delineated reference route in terms of overall shape and principal directions; however, deviations remain in some turning regions and closed-loop structures. In comparison, the trajectories corrected by the CIB method show higher overall consistency with the manually delineated reference route and better geometric coordination in major turning sections and closed-loop structures.

The route-relative deviation metrics further show that the CIB method achieves lower Mean Trajectory Error, RMSE, 95% Error, and Max Error across all three experimental groups. Compared with the uncompensated method, the CIB method reduces the four error metrics by 71.56–77.99%, 68.57–69.56%, and 56.52–64.80% in the three experimental groups, respectively. In addition, the CIB method achieves further error reductions across all four metrics compared with traditional HDE, demonstrating better overall route-relative geometric performance under the evaluated comprehensive-path conditions.

Static zero-bias compensation can only reflect the initial sensor bias measured under static conditions and is difficult to fully represent the overall heading drift caused jointly by gyroscope bias, noise, attitude disturbances, and cumulative integration errors during actual walking. In comparison, the CIB method incorporates the raw reconstructed trajectory, stepwise heading sequence, and trajectory geometric constraints into the reasoning process and progressively determines the equivalent zero-bias compensation parameter through candidate generation, trajectory comparison, and local refinement. It should also be noted that PDR trajectory reconstruction is affected by both step length and heading, and local single-step length errors may cause positional deviations during recursive position updating. However, the uncompensated method, static zero-bias compensation method, HDE, and CIB method used the same single-step length sequence and differed only in their heading compensation strategies. Therefore, the single-step length estimation errors do not affect the relative comparison among the four heading-correction methods.

### 3.5. Repeatability and Output Stability Analysis of LLM-Guided Reasoning

Considering that the reasoning outputs of large language models may exhibit a certain degree of stochasticity, a repeatability test was conducted to further evaluate the output stability of the proposed LLM-guided method under repeated runs. One representative experimental sample was selected from each of the three tasks, namely step-count detection, average step-length estimation, and CIB-based heading correction. For each task, the input data, prompt content, and LLM configuration were kept identical. The same experimental data were independently processed five times without changing any input conditions or introducing manual adjustments, and the output of each run was recorded.

For the step-count detection task, the detected number of steps obtained in each run was recorded. For the average step-length estimation task, the estimated average step length from each run was recorded. For the CIB-based heading correction task, the final equivalent zero-bias compensation parameter determined through the iterative reasoning process was recorded. For continuous numerical outputs, the mean and standard deviation of the five independent runs were further calculated to evaluate the variability of the LLM outputs across repeated reasoning runs. The experimental results are presented in Table 9.

As shown in Table 9, when the input data and prompts were kept identical, the LLM outputs remained generally consistent across five independent runs. Although a certain degree of numerical variation was observed among repeated runs, the outputs for each task remained within a similar range and did not noticeably affect the overall performance assessment or the conclusions of the experiments.

## 4. Discussion

### 4.1. Interpretation of Experimental Findings

The experimental results show that the proposed LLM-guided IMU-PDR method achieved relatively stable performance in step-count detection, step-length estimation, and heading correction under the conditions evaluated in this study. The step-count detection method maintained low errors across different participants, trajectory structures, walking speeds, motion states, and smartphone-carrying configurations. The step-length estimation method achieved competitive average estimation accuracy under both controlled and natural-walking conditions. In addition, the proposed CIB method reduced route-relative trajectory deviations and improved the overall geometric consistency of the reconstructed trajectories. Overall, these results demonstrate the feasibility of incorporating domain-specific prior knowledge into LLM-guided reasoning for key IMU-PDR detection and error-correction tasks.

In terms of step-count detection, the performance degradation of conventional peak-based methods may be mainly associated with their dependence on fixed thresholds, peak–valley pairing rules, and local gait rhythms. Differences in physical characteristics, gait habits, holding postures, and carrying conditions among participants may lead to variations in peak–valley amplitude, peak-shape integrity, and local rhythm in the acceleration signals, making a single fixed parameter setting difficult to apply consistently across participants and walking scenarios. Overall, conventional peak-based methods are sensitive to changes in signal-amplitude distribution and gait rhythm because of their dependence on fixed parameters. In comparison, the LLM-guided step-count detection method incorporates prior knowledge of gait periodicity, peak prominence, rhythm consistency, and peak-shape integrity through prompts and makes decisions by jointly considering multiple signal characteristics, thereby reducing its dependence on fixed thresholds. Under the controlled, natural-walking, stop-and-go, and pocket-carrying conditions evaluated in this study, the proposed method generally maintained low detection errors across different trajectory structures, motion states, and the two evaluated smartphone-carrying configurations.

In terms of step-length estimation, conventional empirical models are generally constrained by predetermined relationships between individual characteristics or acceleration-derived features and step length. The height-based proportional model produces a fixed estimate according to each participant’s height and therefore has difficulty reflecting gait variations and local motion disturbances across different experimental datasets. Although the Weinberg model incorporates dynamic acceleration information, its estimation performance still depends on the correspondence between the empirical coefficient and the current participant, sensor placement, and walking condition. Therefore, when the trajectory structure, gait rhythm, and local motion characteristics change, the applicability of a previously determined parameter may decrease. In comparison, the LLM-guided step-length estimation method combines the observed acceleration characteristics with physical-scale constraints and estimates step length according to the gait information contained in each experimental dataset, thereby reducing its reliance on fixed empirical relationships and manually calibrated coefficients. Under the controlled and natural-walking conditions evaluated in this study, the proposed method demonstrated competitive average step-length estimation accuracy.

In terms of heading correction, among the methods compared in this study, static bias compensation relies on the initial sensor bias measured during the stationary period, whereas traditional HDE relies on predefined heuristic heading constraints. Static bias compensation has difficulty fully representing the accumulated heading drift caused jointly by gyroscope bias, noise, attitude disturbances, and integration errors during actual walking, whereas the effectiveness of HDE may be affected by the correspondence between its predefined heading constraints and the geometric characteristics of the current trajectory. In comparison, the proposed CIB method incorporates the stepwise heading sequence, reconstructed trajectory, and trajectory geometric constraints into the reasoning process and progressively determines an equivalent zero-bias compensation parameter according to the actual drift pattern of each experimental trajectory through candidate generation, trajectory comparison, and local refinement. Under the comprehensive-path conditions evaluated in this study, the CIB method achieved lower route-relative deviations and improved the overall geometric consistency of the reconstructed trajectories, indicating its effectiveness in suppressing accumulated heading drift.

Taken together, the findings from step-count detection, step-length estimation, and heading correction indicate that the primary value of the proposed framework lies in its ability to integrate domain knowledge through a unified prompt-based reasoning framework and to analyze and make judgments according to the characteristics of the current data, thereby reducing reliance on repeated preliminary experiments, manually specified thresholds, and repeated calibration of empirical parameters. Moreover, the method does not require additional training or fine-tuning of the LLM using the current experimental data.

### 4.2. Limitations and Future Directions

The present study represents an initial proof-of-concept investigation of LLM-guided reasoning for IMU-PDR detection and error correction. Its primary objective is to explore the feasibility and potential performance benefits of incorporating domain-specific knowledge into LLM-based reasoning, rather than to develop a lightweight real-time PDR system. Within this scope, several methodological and practical limitations should be acknowledged. First, although this study considered different walking speeds, stop-and-go motion, and two smartphone-carrying configurations—holding the device steadily in front of the waist and carrying it vertically in a trouser pocket—the proposed method has not yet been systematically evaluated under more diverse and challenging gait, motion, and smartphone-usage conditions. These conditions include abnormal gait, irregular walking, more frequent motion-state transitions, handheld smartphone use with natural arm swing, carrying the smartphone in a backpack, making phone calls, and stair ascent and descent. Therefore, future work should conduct broader experiments under these more complex conditions to further evaluate the generalizability and adaptability of the proposed method. In addition, the LLM-guided reasoning mechanism can be further optimized to better handle unstable gait rhythms, enhanced local motion disturbances, and weak trajectory-structure constraints. Second, in terms of trajectory reconstruction accuracy, this paper mainly validates the effectiveness of the LLM-guided method in average step-length estimation. However, the trajectory update process relies on a step-by-step single-step length sequence. Because single-step length may be affected by multiple factors, such as local acceleration fluctuations, gait-cycle variations, sensor attitude disturbances, and environmental factors, ensuring only the accuracy of the average step length is still insufficient to completely avoid local trajectory deviations. Therefore, richer gait information or constraint mechanisms can be further introduced in future work, and the integration of LLM-guided reasoning with HDE, filtering-based fusion, deep learning, and related methods can be explored. Meanwhile, segmented local equivalent zero-bias compensation strategies can be further investigated to achieve more refined step-length estimation, heading-drift correction, and trajectory reconstruction optimization. Third, the comprehensive-path experiment used a manually delineated reference route derived from Google Earth imagery rather than time-synchronized position measurements collected during the experiment. Therefore, the reported trajectory metrics mainly characterize route-relative geometric deviations and do not constitute a validation of the absolute positioning accuracy of the complete system. Future work should introduce time-synchronized positional ground truth to enable a more rigorous evaluation of trajectory reconstruction and absolute localization accuracy. Fourth, to minimize potential contextual interference across experiments, each experimental task was conducted in a separate conversation, while the input data and prompt settings for the corresponding task were kept consistent. Given the stochastic nature of LLM outputs, additional runs were performed in a limited number of cases when the initial outputs exhibited relatively large deviations, and one of the repeated outputs was selected for subsequent analysis. The numerical values generated by the LLM were not manually modified by the authors. Examination of the repeated runs showed that the differences among the outputs were generally small and did not affect the overall experimental conclusions. Nevertheless, because this procedure did not follow a predefined and standardized rerun or output-selection protocol, it may have introduced a certain degree of selection bias, which is acknowledged as a limitation of the current study.

Beyond these methodological limitations, the practical deployment of the current LLM-guided framework also requires further consideration of real-time feasibility, processing latency, and dependence on online services.

The current implementation of the proposed LLM-guided IMU-PDR framework serves as an offline proof of concept rather than a real-time localization system. In this study, the complete IMU sequence for each experimental trial was first collected, after which data preprocessing, LLM-guided reasoning, heading correction, and trajectory reconstruction were performed offline. Therefore, the current implementation does not support step-by-step online trajectory updates during walking.

To provide an empirical indication of practical processing latency, the end-to-end processing times of representative LLM-guided tasks were manually recorded. For step-count detection, fixed 50-step experimental segments required 34.49 s, 47.49 s, and 33.51 s, with an average processing time of approximately 38.50 s. For average step-length estimation, fixed 50 m experimental segments required 72.13 s, 98.83 s, and 92.23 s, with an average of approximately 87.73 s. For CIB-based heading correction, IMU data collected along an approximately 540 m comprehensive path were processed offline, yielding end-to-end processing times of 201.35 s, 211.74 s, and 233.52 s, with an average of approximately 215.54 s. Because the input lengths, data representations, and reasoning procedures differed across tasks, these processing times are intended only to provide representative end-to-end interaction latencies under the corresponding experimental settings and should not be interpreted as a direct comparison of computational efficiency among tasks. Moreover, the reported measurements represent the complete interaction latency observed through the ChatGPT web interface rather than the standalone inference time of the underlying LLM and may also be influenced by network conditions, server load, input length, and task complexity.

The current framework processes complete experimental sequences offline and performs LLM reasoning through the online ChatGPT service. Therefore, compared with conventional lightweight and self-contained PDR algorithms, the proposed method entails greater processing latency and resource requirements and does not currently support step-by-step online localization. At present, the method is more suitable for offline trajectory analysis and post-processing correction. In practical applications, a potential deployment scenario is a server-assisted PDR framework, in which lightweight on-device PDR algorithms provide continuous localization, while the LLM is invoked on demand on the server side for PDR analysis and accumulated heading-drift correction, thereby shifting part of the computational burden away from resource-constrained devices. Future work will investigate automated interaction workflows, more efficient reasoning strategies, and lightweight or locally deployable models to improve the practicality of server-assisted implementation and explore the feasibility of fully local real-time deployment on edge devices.

## 5. Conclusions

This paper proposes an LLM-guided IMU-PDR detection and correction method to improve step-count detection, step-length estimation, and heading-drift correction in pedestrian dead reckoning. The proposed method reduces the dependence on fixed thresholds and empirical coefficients in step-count detection and step-length estimation and avoids the limitation of directly using static zero bias as the sole compensation basis in heading correction, thereby improving its adaptability in solving PDR tasks under different scenarios. Under controlled experimental conditions, the proposed LLM-guided step-count detection method achieved stable step-count detection results across three participants and three typical trajectories, with an average error of 2.84%, which was lower than those of the peak-valley pairwise detection method, fixed double-threshold peak detection method, and ATT-EGP method. The natural-walking, stop-and-go, and pocket-carrying experiments further showed that the proposed method achieved average step-count errors of approximately 0.56%, 2.45%, and 1.11%, respectively, demonstrating its applicability under less constrained and more complex walking conditions. The LLM-guided step-length estimation method achieved an error range of 0.53–4.18%, with an average error of approximately 2.51%, under the controlled experimental conditions, avoiding repeated parameter tuning for different participants and experimental scenarios while exhibiting good stability and usability. Under natural walking conditions without predefined footfall markings, the proposed method achieved an average relative error of approximately 0.61%, further demonstrating its applicability to average step-length estimation under natural gait conditions. For heading correction, this paper further proposes the CIB method, which effectively reduced route-relative trajectory reconstruction errors and improved trajectory geometric consistency compared with the uncompensated method, static zero-bias compensation, and HDE. Across the three experimental groups, the error reduction range relative to the uncompensated method was approximately 56.52–77.99%, demonstrating its advantages in reducing overall trajectory deviation, large local errors, and extreme deviations, as well as improving the geometric consistency of reconstructed PDR trajectories. Nevertheless, the present study remains limited by the range of evaluated gait and smartphone-use conditions, the emphasis on average rather than stepwise step-length validation, the use of a manually delineated reference route instead of time-synchronized positional ground truth, and the current offline web-based implementation and the non-standardized rerun and output-selection procedure. Future work should address these issues to further establish the generalizability, trajectory-level validity, and practical deployability of the proposed method.

Overall, despite these limitations, the research results indicate that, under the guidance of domain prior knowledge and task constraints, LLMs have the potential to structurally interpret and reason over IMU signals, providing a new technical pathway for intelligent detection and error correction in IMU-PDR.

## Figures and Tables

**Table 1:** Sensor parameters.

Scheme	Range	Resolution	Sampling Rate
Accelerometer	±8 g	0.00239 m/s^2^	100 Hz
Gyroscope	±2000°/s	0.00122 rad/s	100 Hz

**Table 2:** Basic participant information.

Participant	Sex	Age (Years)	Height (m)	Weight (kg)
P1	Male	21	1.83	88
P2	Female	47	1.68	63
P3	Male	48	1.74	81

**Table 3:** Step-count detection results under controlled experimental conditions.

Participant	Trajectory	True Steps	LLM Avg. Det./Err. (%)	PVP Avg. Det./Err. (%)	FDT Avg. Det./Err. (%)	ATT-EGP Avg. Det./Err. (%)
P1	Straight	50	50.00/0.00	52.00/4.00	50.50/1.00	50.25/0.50
P1	Rectangular	92	95.25/3.53	105.25/14.40	99.25/7.88	97.75/6.25
P1	S-shaped	41	41.75/1.83	40.50/1.22	44.75/9.15	42.25/3.05
P2	Straight	50	51.50/3.00	51.50/3.00	51.00/2.00	51.00/2.00
P2	Rectangular	88	90.33/2.65	90.33/2.65	94.67/7.58	92.33/4.92
P2	S-shaped	41	42.50/3.66	34.50/15.85	43.00/4.88	41.50/1.22
P3	Straight	50	51.50/3.00	55.00/10.00	51.25/2.50	51.25/2.50
P3	Rectangular	88	91.33/3.78	78.00/11.36	92.00/4.55	91.00/3.41
P3	S-shaped	41	42.67/4.07	23.67/42.27	41.67/1.63	21.67/47.15

Note: Avg. Det./Err. (%) denotes the average detected step count and relative error. The compared methods include the proposed large language model-guided step-count detection method (LLM), the peak-valley pairwise step-count detection method (PVP), the fixed double-threshold peak detection method (FDT), and the adaptive time-threshold detection method based on the estimated gait period (ATT-EGP).

**Table 4:** Step-length estimation results under controlled conditions.

Participant	Trajectory	Ref. SL (m)	LLM Est. SL (m)/Err. (%)	HBPM Est. SL (m)/Err. (%)	Weinberg Est. SL(m)/Err. (%)
P1	Straight	0.76	0.765/0.66	0.759/0.13	0.760/0.00
P1	Rectangular	0.76	0.753/0.92	0.759/0.13	0.741/2.50
P1	S-shaped	0.75	0.754/0.53	0.759/1.20	0.710/5.33
P2	Straight	0.67	0.692/3.28	0.697/4.03	0.671/0.15
P2	Rectangular	0.67	0.698/4.18	0.697/4.03	0.640/4.48
P2	S-shaped	0.67	0.696/3.88	0.697/4.03	0.653/2.54
P3	Straight	0.70	0.717/2.43	0.722/3.14	0.701/0.14
P3	Rectangular	0.70	0.727/3.86	0.722/3.14	0.651/7.00
P3	S-shaped	0.70	0.720/2.86	0.722/3.14	0.638/8.86

Note: SL denotes step length. Ref. SL and Est. SL denote the reference step length and estimated step length, respectively. Err. denotes the relative error. The compared methods include the proposed large language model-guided step-length estimation method (LLM), the height-based proportional model (HBPM), and the Weinberg nonlinear step-length model (Weinberg).

**Table 5:** Step-count detection results under natural walking conditions.

Participant	Speed	True Steps	LLM Avg. Det./Err. (%)	PVP Avg. Det./Err. (%)	FDT Avg. Det./Err. (%)	ATT-EGP Avg. Det./Err. (%)
P1	Normal	50	50.00 ± 0.00/0.00	51.00 ± 1.00/2.00	50.67 ± 0.58/1.34	50.67 ± 0.58/1.34
P2	Normal	50	50.67 ± 0.58/1.34	49.67 ± 1.15/0.66	51.00 ± 1.00/2.00	51.00 ± 1.00/2.00
P3	Normal	50	50.00 ± 0.00/0.00	64.67 ± 7.51/29.34	51.33 ± 0.58/2.66	51.67 ± 0.58/3.34
P1	Fast	50	50.00 ± 0.00/0.00	50.33 ± 0.58/0.66	50.67 ± 1.15/1.34	50.67 ± 1.15/1.34
P2	Fast	50	51.00 ± 1.00/2.00	51.00 ± 1.00/2.00	52.00 ± 0.00/4.00	52.00 ± 0.00/4.00
P3	Fast	50	50.00 ± 0.00/0.00	54.00 ± 1.73/8.00	51.33 ± 0.58/2.66	51.33 ± 0.58/2.66

Note: Avg. Det. denotes the average detected step count, and Err. denotes the relative error. Detected step counts are reported as the mean ± sample standard deviation across three independent trials, followed by the relative error (%), which was calculated from the reported mean according to Equation (4).

**Table 6:** Results of step-length estimation experiments under natural walking conditions.

Participant	Trajectory	Ref. SL (m)	LLM Est. SL (m)/Err. (%)	HBPM Est. SL (m)/Err. (%)	Weinberg Est. SL(m)/Err. (%)
P1	Straight	0.754 ± 0.0065	0.757 ± 0.0044/0.40	0.759 ± 0.0000/0.66	0.771 ± 0.0043/2.25
P2	Straight	0.694 ± 0.0000	0.699 ± 0.0194/0.72	0.697 ± 0.0000/0.43	0.667 ± 0.0119/3.89
P3	Straight	0.714 ± 0.0102	0.709 ± 0.0081/0.70	0.722 ± 0.0000/1.12	0.771 ± 0.0151/7.98

Note: SL denotes step length. Ref. SL and Est. SL denote the reference and estimated step lengths, respectively, and Err. denotes the relative error. Reference step lengths are presented as mean ± sample standard deviation, whereas estimated step lengths are presented as mean ± sample standard deviation/relative error (%). Relative errors were calculated from the reported mean estimated and reference step lengths.

**Table 7:** Results of step-count detection experiments under intermittent walking and stopping conditions.

Participant	Condition	True Steps	LLM Avg. Det./Err. (%)	PVP Avg. Det./Err. (%)	FDT Avg. Det./Err. (%)	ATT-EGP Avg. Det./Err. (%)
P1	Stop-and-go	50	50.67 ± 1.15/1.34	53.00 ± 2.65/6.00	52.33 ± 0.58/4.66	51.33 ± 0.58/2.66
P2	Stop-and-go	50	51.33 ± 2.31/2.66	49.00 ± 2.00/2.00	52.00 ± 1.73/4.00	51.67 ± 2.31/3.34
P3	Stop-and-go	50	51.67 ± 2.89/3.34	67.00 ± 7.00/34.00	55.33 ± 0.58/10.66	55.33 ± 0.58/10.66

**Table 8:** Results of step-count detection experiments under pocket-carrying conditions.

Participant	Condition	True Steps	LLM Avg. Det./Err. (%)	PVP Avg. Det./Err. (%)	FDT Avg. Det./Err. (%)	ATT-EGP Avg. Det./Err. (%)
P1	Pocket	50	50.67 ± 1.53/1.34	66.00 ± 4.00/32.00	69.33 ± 5.69/38.66	72.67 ± 6.03/45.34
P2	Pocket	50	49.33 ± 1.15/1.34	60.33 ± 0.58/20.66	59.00 ± 6.08/18.00	60.67 ± 4.73/21.34
P3	Pocket	50	49.67 ± 0.58/0.66	97.67 ± 5.77/95.34	64.67 ± 3.06/29.34	98.00 ± 1.00/96.00

**Table 9:** Repeatability results of the LLM-guided method.

Task	Run1	Run2	Run3	Run4	Run5	Mean ± SD
Step detection	50	50	50	50	50	50.00 ± 0.00
Step-length estimation	0.759	0.758	0.758	0.759	0.759	0.7586 ± 0.0005
CIB equivalent bias	−0.00270	−0.00275	−0.00270	−0.00300	−0.00285	−0.002800 ± 0.000127

## Data Availability

The data presented in this study are available from the corresponding author upon reasonable request, subject to privacy and ethical restrictions.

## Appendix A

**Table A1:** Example experimental prompts.

Task Type	Example Prompt
Step-count detection	You are an IMU-based gait-recognition expert. During walking, the gravity-removed three-axis resultant acceleration exhibits a stable periodic peak-valley structure, and each true peak corresponds to one step. The data may contain noise impulses, device shaking, and local abrupt changes; therefore, pseudo-peaks should be removed. True gait peaks should satisfy the following physical rules: periodicity, meaning that peak intervals should satisfy a reasonable gait-frequency constraint and should not be overly dense; prominence, meaning that the peak amplitude should be significantly higher than the local baseline; rhythmicity, meaning that consecutive peaks should maintain approximately stable step intervals and abnormal isolated peaks should be excluded; and peak-shape consistency, meaning that the peak shape should be smooth and complete, whereas sharp or distorted peaks should be regarded as pseudo-peaks. Given the dynamic acceleration time series, identify true gait peaks according to the above rules and output the final step count. Do not explain the reason; output only one integer.
Step-length estimation	You are an IMU-based gait-analysis expert. Please read and parse the file I provide. The file contains a gravity-removed waist-level single-channel acceleration time series (unit: g; sampling rate: 100 Hz) corresponding to a stable walking process. This signal can reflect the vertical oscillation characteristics of the human center of mass during gait. In this stable walking segment, the step cycles exhibit good consistency. Please identify the true gait cycles according to the periodic variation pattern of the signal, and extract effective fluctuation features from each step cycle that characterize single-step motion intensity. On this basis, estimate the length of each step according to the reasonable correspondence between these features and step length. The estimation results should reflect that the step length increases as the feature becomes stronger, but the growth trend gradually saturates rather than changing in a simple linear manner. Finally, perform consistency statistics on the step lengths corresponding to the whole signal and output the average step length (unit: m). The subject’s height is known to be 1.83 m; this information is used only to constrain the physical scale of the result. Do not explain the process or reason; output only one numerical value.
CIB heading correction	Given the stepwise heading sequence and its corresponding raw reconstructed trajectory, identify whether each local segment belongs to straight walking, continuous turning, in-place turning, or abnormal fluctuation. Then, based on the number of straight segments, the distribution of turning segments, parallel relationships, turning continuity, and closed-loop tendency, infer the most likely target spatial structure before trajectory drift. The overall structure should be determined primarily according to local heading variations and segment-connection relationships, rather than the visual appearance of the current trajectory. Care should be taken not to be misled by bending, tailing, opening, or compression caused by residual bias. Output only: local motion structure identification and overall structure detection. Based on the results from the previous stage, analyze the local and global drift patterns caused by residual bias in the current trajectory, including bending of straight segments, tailing of turning segments, main-direction deviation, weakened parallel relationships, and insufficient loop closure. Then, extract the target geometric constraints that should be satisfied by subsequent compensation. Output only: local and global drift analysis and target geometric constraints. Based on the above analysis, the gyroscope z-axis zero bias measured under static conditions is known to be −0.00039 rad/s. This value is used only as a reference. To avoid misjudging the compensation direction and search interval, the initial exploration candidates should cover negative compensation, near-zero compensation, and positive compensation, while maintaining a sufficiently large interval between adjacent candidates to include both clear under-compensation and clear over-compensation cases. This process is used to preliminarily determine the empirical effective interval. Output only: equivalent zero-bias compensation direction judgment, seven initial exploration candidate values, and boundary exploration basis. Determine the effective interval based on the above candidate compensation values and their corresponding trajectories. Sort the candidates in ascending order of the compensation value, and determine whether each candidate corresponds to under-compensation, a near-optimal case, or over-compensation. Note that not all three cases necessarily appear among the candidates. The effective interval should be jointly bounded by the nearest clearly under-compensated candidate and the nearest clearly over-compensated candidate on both sides of the near-optimal region. The candidate state should be judged only according to the target geometric structure, rather than the sign or numerical magnitude of the compensation value. The direction of compensation and whether a candidate corresponds to under-compensation or over-compensation should not be inferred from the compensation value itself. The effective interval is valid only when clear geometric degradation occurs on both sides of the near-optimal region, and the degradation directions are different. Specifically, one side should correspond to unresolved original drift, whereas the other side should correspond to reverse degradation caused by excessive compensation. A candidate should not be judged as over-compensated merely because its trajectory shape becomes worse. If the overall trajectory becomes worse but the degradation pattern remains similar, the boundary has not yet been determined. If the boundary on one side is unclear, continue expanding the exploration toward that side and provide the corresponding candidates. Local refinement should be performed only after both boundaries are clearly determined. Based on the initial exploration candidate values and their corresponding trajectories, determine the current empirical effective interval and regenerate six candidate equivalent zero-bias compensation parameters within this interval. Output only: current empirical effective interval judgment, six candidate parameters, and the basis for candidate-parameter selection. Compare the corrected trajectories corresponding to the six candidate parameters from the previous round, determine the local near-optimal region containing the current optimal candidate, and further generate four finer candidate parameters around it. Output only: comparison results of the six candidate trajectories, current optimal candidate judgment, and four finer candidate parameters. Compare the corrected trajectories corresponding to the four refined candidate parameters and determine the final equivalent zero-bias compensation parameter by comprehensively considering local motion continuity, overall structural coordination, and the degree to which the geometric constraints are satisfied. Output only: final comparison results of the four refined candidate trajectories, final equivalent zero-bias compensation parameter judgment, and final selection basis.

## Appendix B

### Appendix B.1. Representative Input Example for Step-Count Detection

Representative excerpt of the gravity-removed three-axis resultant acceleration sequence used for step-count detection.

0.0827450020255449

−0.00598863479609579

−0.030141919814586

−0.0687033570531699

−0.052979622028131

### Appendix B.2. Representative Input Example for Step-Length Estimation

Representative excerpt of the gravity-removed waist-level single-axis acceleration sequence used for step-length estimation.

0.011242244897959

0.0182010204081633

0.0247629591836733

0.0288831632653059

0.0218939795918367

### Appendix B.3. Representative Input Example for CIB-Based Heading Correction

For CIB-based heading correction, the .mat file containing the stepwise heading-related data was provided to the LLM together with the corresponding two-dimensional trajectory visualizations reconstructed in MATLAB. A representative excerpt of the stepwise heading sequence contained in the .mat file and an example of the corresponding two-dimensional trajectory visualization are shown below.

0.0284

−0.0263

0.0889

0.0073

0.0622
